# Clinical Characteristics and Management Outcomes of Severe Multi-Valvular Rheumatic Heart Disease: A Hospital-Based Analysis

**DOI:** 10.7759/cureus.106946

**Published:** 2026-04-13

**Authors:** Shraiya Stan, Ananjan Bhattacharya

**Affiliations:** 1 Accident and Emergency, Lal Bahadur Shastri Hospital, Delhi, IND; 2 Accident and Emergency, Cantonment General Hospital, New Delhi, IND

**Keywords:** atrial fibrillation, healthcare-associated infections, multi-valvular disease, pulmonary hypertension, rheumatic heart disease

## Abstract

Background: Multi-valvular involvement represents the most severe form of rheumatic heart disease (RHD), presenting complex clinical challenges requiring a comprehensive understanding for optimal patient management. In resource-limited settings, late presentation and inadequate secondary prophylaxis result in advanced disease with high complication burdens.

Objective: To analyze the clinical presentation, diagnostic findings, treatment strategies, and outcomes, specifically in-hospital complication rates, mortality, functional improvement, and six-month follow-up outcomes in patients with severe multi-valvular RHD presenting to secondary and tertiary academic centers in Delhi, India.

Methods: A retrospective observational analysis was conducted of 385 consecutive patients with severe multi-valvular RHD admitted to Lal Bahadur Shastri Hospital and Dr. Ram Manohar Lohia Hospital, New Delhi, between September 2023 and September 2025. Clinical data, echocardiographic parameters, laboratory findings, treatment protocols, and outcomes were systematically reviewed and analyzed.

Results: Mean age was 31.2 ± 7.4 years; 68% were female. The majority (57%) presented with New York Heart Association Functional Classification (NYHA) Class III-IV symptoms. Atrial fibrillation was documented in 58%, severe pulmonary hypertension (>60 mmHg) in 82%, and right ventricular dysfunction in 69%. Healthcare-associated infections occurred in 23%, including MRSA bacteremia in 12%. In-hospital mortality was 3.4%; 6-month mortality was 7.2%. Admissions peaked during monsoon months (18% higher case volume).

Conclusion: Severe multi-valvular rheumatic heart disease (RHD) presents complex management challenges requiring comprehensive evaluation and multidisciplinary care. Early recognition of complications and timely intervention are crucial for improving outcomes in this high-risk population.

## Introduction

Rheumatic heart disease (RHD) continues to be a major cause of cardiovascular morbidity and mortality in developing countries, affecting approximately 33 million people worldwide with an annual mortality rate of 275,000 [1]. The disease results from an autoimmune response to group A streptococcal infection, leading to progressive valvular damage that predominantly affects the mitral valve, followed by aortic valve involvement [2]. 

Multi-valvular RHD represents the most severe form of the disease, associated with significantly higher mortality rates compared to single-valve involvement [3]. The complex hemodynamic alterations resulting from multiple valve lesions create challenging clinical scenarios that require sophisticated management strategies. Recent studies have shown that multi-valvular RHD carries a five-year mortality rate approximately 40% higher than single-valve disease without surgical intervention [4].

The progression from acute rheumatic fever to chronic RHD typically occurs over decades, but accelerated disease courses have been documented in regions with limited healthcare access [5]. The combination of mitral stenosis and regurgitation, often accompanied by tricuspid involvement, creates particularly challenging hemodynamic conditions that frequently lead to pulmonary hypertension and right heart failure. 

Contemporary management of severe multi-valvular RHD involves complex decision-making regarding medical optimization, timing of surgical intervention, and management of complications such as arrhythmia and infections [6]. 

Despite the growing literature on single-valve RHD, granular data on multi-valvular involvement remain scarce from tertiary academic centers in Delhi. Specifically, data on seasonal variation in complication patterns, contemporary infectious complication profiles (including drug-resistant organisms such as methicillin-resistant *Staphylococcus aureus* [MRSA]), and echocardiographic severity distribution in this subpopulation are limited from this region. Existing studies have predominantly focused on mitral stenosis alone or have not reported multi-season complication data. This study addresses this gap by providing a comprehensive real-world characterization of severe multivalvular RHD in a Delhi-based secondary and tertiary academic setting, generating descriptive data to inform region-specific management protocols and public health priorities.

## Materials and methods

Study design and setting

This retrospective observational study was conducted at two academic medical centers in Delhi, India: Lal Bahadur Shastri Hospital, a government-run teaching hospital, and Dr. Ram Manohar Lohia Hospital, a tertiary hospital with a dedicated cardiac referral center having active departments of cardiology, cardiac surgery, and cardiac anesthesiology and together serve as primary referral destinations for complex cardiac cases from Delhi and surrounding NCR regions. Clinical data from patients admitted between September 2023 and September 2025 were analyzed. It serves as the primary and often sole tertiary cardiac referral center for a broad urban and peri-urban catchment area spanning multiple districts of Delhi NCR, where the limited availability of local cardiac surgical infrastructure means that most patients with symptomatic, complex multi-valvular RHD are referred here. While this implies that our cohort represents advanced disease, it is likely broadly representative of the spectrum of multi-valvular RHD requiring tertiary-level care in this region.

Study population

The study included patients aged 18-50 years with a confirmed diagnosis of severe multi-valvular RHD with involvement of at least two cardiac valves with severe dysfunction, complete clinical and echocardiographic data, and hospital admission ≥7 days. Exclusion criteria: congenital heart disease, previous cardiac surgery, acute rheumatic fever without established chronic valvular disease, incomplete records, or primarily degenerative valve disease.

The minimum seven-day stay criterion ensured adequate data completeness for retrospective analysis, as shorter admissions frequently lacked complete echocardiographic workup and outcome documentation. We acknowledge two potential biases: (1) exclusion of milder cases managed with early discharge and (2) exclusion of rapidly fatal cases that died before accumulating seven inpatient days. Complication rates and mortality figures should therefore be interpreted as representative of the hospitalized, clinically complex multi-valvular RHD population rather than the full disease spectrum.

Sample size justification

A minimum sample size of 385 patients was determined to estimate prevalence outcomes with acceptable precision. The sample size was calculated using the standard formula for estimating a proportion [7]: n = Z² × p × (1 − p) / d².

Because this was a retrospective study, all consecutive eligible patients during the study period were included, regardless of a pre-specified sample target. As a post-hoc adequacy assessment, the achieved sample (n = 385) was verified as sufficient to estimate key prevalence outcomes with ±5% absolute precision at 95% confidence (n = Z² × p × (1 − p) / d² = 384.16, rounded to 385; Z = 1.96, p = 0.50, d = 0.05 [7]). This confirms dataset adequacy for descriptive objectives but does not confer inferential power for hypothesis testing.

Data collection

Clinical data were extracted from electronic medical records using standardized forms. Variable categories and their descriptive analytic roles were as follows: demographic and socioeconomic variables (age, sex, origin, education, socioeconomic status) to characterize population distribution; clinical history (rheumatic fever history, throat infections, symptom duration) to describe disease chronicity and missed prophylaxis opportunities; functional status, NYHA classification [8], assigned by the admitting cardiologist at initial assessment, used to stratify symptom severity at presentation and discharge; physical examination and ECG findings to describe prevalence of key clinical signs and rhythm abnormalities; diagnostic variables (echocardiographic parameters, inflammatory markers, biomarkers, microbiology) as primary endpoints for characterizing disease severity and complications; and treatment and outcome variables to describe management and its temporal association with clinical course. All variables are reported descriptively only; no inferential or regression models were applied.

Echocardiographic assessment

All patients underwent comprehensive transthoracic echocardiography performed by experienced cardiologists using standardized protocols [9]. The assessment included evaluation of left ventricular dimensions and systolic function, measurement of left atrial size, detailed valvular assessment with severity grading according to established guidelines [10], estimation of pulmonary artery pressure using tricuspid regurgitant jet velocity, evaluation of right ventricular function, and calculation of the Wilkins score for mitral valve morphology assessment [11].

Laboratory analysis

Standard laboratory investigations were performed for all patients and included complete blood count and basic metabolic panel [12], inflammatory markers such as C-reactive protein and erythrocyte sedimentation rate [13], cardiac biomarkers including N-terminal pro-brain natriuretic peptide [14], coagulation studies for anticoagulation monitoring [15], and blood cultures with antimicrobial susceptibility testing [16]. These references reflect widely accepted standards for laboratory testing and interpretation in cardiovascular and infectious disease practice.

Statistical analysis

Descriptive statistics were used to summarize all data (IBM Corp. Released 2020. IBM SPSS Statistics for Windows, Version 26. Armonk, NY: IBM Corp.). Continuous variables were assessed for normality using the Shapiro-Wilk test and expressed as mean ± SD or median (IQR) as appropriate. Categorical variables were presented as frequencies and percentages. Missing data were handled by complete-case analysis; variables with >10% missing values are reported with their effective denominators. No inferential statistical testing, regression modeling, or survival analysis was performed; all findings are strictly descriptive and do not establish associations or causality.

Ethical considerations

This study was conducted in accordance with institutional guidelines and ethical standards for medical research [17]. As this was a retrospective analysis of anonymized clinical data without patient identification, individual informed consent was not required. The study protocol was reviewed and approved by the institutional ethics committee prior to data collection and analysis.

## Results

Patient demographics and clinical characteristics

A total of 385 patients with severe multi-valvular rheumatic heart disease were included in the study. The mean age was 31.2 ± 7.4 years, and the majority were female (68%). Most patients (72%) were of South Asian origin, reflecting the regional demographic pattern. Nearly half (45%) reported a history of recurrent throat infections during childhood, although only 23% had a documented diagnosis of acute rheumatic fever. Approximately 31% had been previously told they had a heart murmur but were lost to follow-up, and the mean duration of cardiac symptoms before presentation was 18.3 ± 12.7 months.

Monthly admission patterns demonstrated a seasonal variation, with peak admissions occurring during the monsoon months (July-September), representing an 18% higher case volume compared to other seasons. The geographic distribution revealed that 45% of patients were from urban areas, 35% from semi-urban areas, and 20% from rural areas. Socioeconomic assessment indicated that 62% of patients belonged to lower-middle or below-poverty-line categories. Regarding educational background, 34% of patients had primary education or less, 41% had secondary education, and 25% had higher education.

Functional status and presenting symptoms

**Figure 1:**
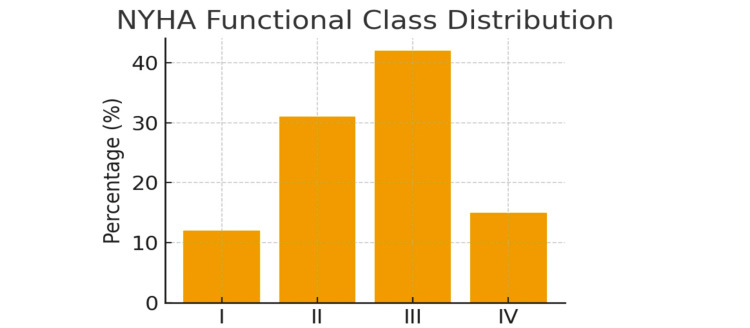
NYHA functional class distribution in severe multi-valvular rheumatic heart disease This bar chart illustrates the functional status distribution among 385 patients with severe multi-valvular rheumatic heart disease at hospital admission. The New York Heart Association (NYHA) functional classification was used to assess symptom severity and exercise tolerance. Class I represents asymptomatic patients with no limitation of physical activity; Class II indicates slight limitation with comfortable activity at rest but symptoms during ordinary physical activity; Class III shows marked limitation with comfortable activity at rest but symptoms during less than ordinary activity; and Class IV represents severe limitation with symptoms at rest. Most patients (57%) presented with advanced symptoms (Class III-IV), reflecting the severe nature of multivalvular disease and late presentation common in resource-limited settings. Data are presented as percentages of the total study population (n=385).

At admission, 12% of patients were classified as NYHA class I, 31% as class II, 42% as class III, and 15% as class IV (Figure 1). The most frequent presenting symptom was dyspnea on exertion (89%), followed by palpitations (76%), peripheral edema (67%), chest pain (34%), and syncope or presyncope (23%).

Physical examination and electrocardiography

On physical examination, 94% had an auscultatory mitral stenosis murmur, 78% had a tricuspid regurgitation murmur, 71% showed elevated jugular venous pressure, 65% exhibited pulmonary crackles, and 67% presented with peripheral edema. Irregular pulse consistent with atrial fibrillation was observed in 58% of cases. Electrocardiography demonstrated atrial fibrillation in 58%, right ventricular hypertrophy in 73%, left atrial enlargement in 31%, right axis deviation in 45%, and a prolonged PR interval in 22%.

Echocardiographic parameters

**Figure 2:**
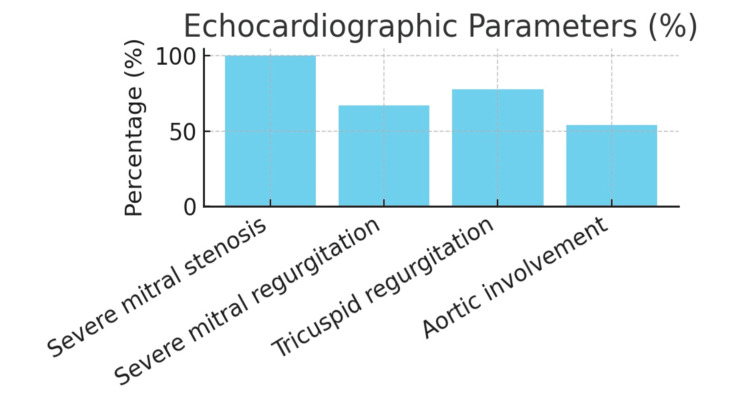
Frequency of valvular involvement on echocardiography in severe multi-valvular rheumatic heart disease This horizontal bar chart demonstrates the prevalence of different valvular involvements identified through comprehensive transthoracic echocardiography in 385 patients with severe multi-valvular rheumatic heart disease. All patients met the inclusion criterion of severe mitral stenosis (valve area <1.5 cm²) as the primary lesion. Valve involvement was classified according to established echocardiographic guidelines, with severe regurgitation defined as effective regurgitant orifice area ≥40 mm² for the mitral valve and ≥30 mm² for the tricuspid valve. Moderate involvement was defined as an effective regurgitant orifice area of 20-39 mm² for the mitral valve and 20-29 mm² for the tricuspid valve. The predominant pattern shows universal mitral stenosis with frequent concurrent regurgitation, emphasizing the complex hemodynamic burden characteristic of advanced rheumatic heart disease. Data are presented as percentages of the total study population (n=385).

Mean left atrial diameter: 48.7 ± 8.2 mm; LVEF: 56.3 ± 8.9%; LVEDD: 46.2 ± 6.1 mm. All patients had severe mitral stenosis (valve area <1.5 cm²). Prevalence at admission echocardiography: severe mitral regurgitation 67%, moderate-to-severe tricuspid regurgitation 78%, mild-to-moderate aortic involvement 54% (Figure 2, prevalence of valvular lesions on admission echocardiography). Mean Wilkins score: 9.2 ± 2.1. Severe pulmonary hypertension (>60 mmHg): 82%; right ventricular dysfunction: 69%; mean TAPSE: 15.1 ± 3.2 mm.

Descriptive cross-tabulation: Among patients with atrial fibrillation (n = 223, 58%), severe pulmonary hypertension was observed in 87%, compared to 75% among those in sinus rhythm, a descriptive difference noted without inferential testing. NYHA Class III-IV patients had a mean hospital stay of 21.4 ± 11.2 days, compared to 12.3 ± 4.7 days for Class I-II patients. These observations are reported as descriptive patterns only and do not establish causal or predictive relationships.

Laboratory findings

Inflammatory markers were frequently elevated, with 71% of patients showing C-reactive protein above 0.5 mg/dL and 65% having an elevated erythrocyte sedimentation rate. The mean NT-proBNP was 2,847 ± 1,623 pg/mL. Most patients (89%) had normal serum creatinine, but 34% had mild elevation in liver enzymes, and 45% showed imaging evidence of hepatic congestion.

Complications and comorbidities

Cardiovascular complications were common, including atrial fibrillation in 58%, heart failure with preserved ejection fraction in 73%, pulmonary hypertension in 82%, and right heart failure in 69%. Infectious complications were also frequent: 23% developed healthcare-associated infections, including MRSA bacteremia in 12%, respiratory tract infections in 31%, and urinary tract infections in 15%. Other complications included acute kidney injury (19%), thrombocytopenia (8%), and hepatic congestion (45%).

Treatment strategies

Most patients received comprehensive medical therapy. Rate control for atrial fibrillation was employed in 58%, anticoagulation in 73%, diuretic therapy in 89%, ACE inhibitors or ARBs in 67%, and beta-blockers in 71%. Prophylactic antibiotics were used in 34%, and 23% required treatment of active infections. Vancomycin was administered to 12% of patients for MRSA, and combination antimicrobial therapy was used in 15%. Advanced supportive measures included mechanical ventilation (11%), vasopressor support (8%), and central venous monitoring (19%).

Hospital course and outcomes

The mean hospital stay was 16.7 ± 8.9 days, with 23% requiring ICU admission for a mean of 4.2 ± 2.8 days. In-hospital mortality was 3.4%. At discharge, 78% of patients demonstrated improved functional class, 89% were hemodynamically stable, 91% had resolution of active infections, and 65% had a planned surgical intervention. Major complications during hospitalization included acute decompensation in 27%, nosocomial infections in 23%, arrhythmias in 31%, and renal dysfunction in 19%.

Seasonal variations and quality metrics

Seasonal analysis revealed distinct patterns of complications. The winter months (December-February) were associated with a higher incidence of respiratory complications (38% compared to 24% during other seasons). The summer months (April-June) showed an increased frequency of dehydration-related complications (15% compared to 8%), while the monsoon season (July-September) demonstrated a peak in infectious complications (31% compared to 19%). Quality metrics indicated a mean door-to-echocardiography time of 4.2 ± 2.1 hours and a mean time to appropriate anticoagulation of 12.7 ± 6.3 hours. Length of stay varied according to disease severity, with New York Heart Association (NYHA) Class I-II patients averaging 12.3 ± 4.7 days compared to 21.4 ± 11.2 days for NYHA Class III-IV patients.

Follow-up outcomes

Six-month follow-up data were available for 67% of discharged patients (n = 248). The readmission rate within six months was 23%, while sustained functional improvement was documented in 71% of patients. Surgical intervention was completed in 42% of patients during the follow-up period. Extended mortality at 6-month follow-up was 7.2%.

## Discussion

The following discussion is based entirely on descriptive, observational data from a retrospective cohort. No causal inferences can be drawn. All findings should be interpreted in the context of the study limitations detailed below.

Valvular pathology and hemodynamic consequences

This study describes a pattern of severe multi-valvular RHD characterized by universal mitral stenosis with frequent concurrent regurgitation, tricuspid involvement in 78%, and severe pulmonary hypertension in 82% at presentation, consistent with advanced disease stages reported in prior literature from resource-limited settings. The high observed prevalence of severe pulmonary hypertension is consistent with the pattern of late presentation and prolonged chronicity in this cohort (mean symptom duration 18.3 months). These are cross-sectional observations; prospective evaluation would be needed to determine whether earlier intervention alters pulmonary vascular outcomes.

Arrhythmias and thromboembolic risk

Atrial fibrillation was observed in 58% of patients, consistent with the degree of left atrial enlargement documented (mean 48.7 mm). Anticoagulation was employed in 73%, reflecting the high thromboembolic risk profile of this population. The relationship between anticoagulation use and clinical outcomes cannot be assessed from these descriptive data alone.

Infectious complications

Healthcare-associated infections occurred in 23% of patients (per CDC/NHSN criteria), including MRSA bacteremia in 12%, substantially higher than rates in general cardiology populations. This pattern is descriptively consistent with prolonged hospitalization (mean: 16.7 days), frequent invasive monitoring (19%), and chronic disease burden. These observations highlight the potential importance of structured infection prevention protocols, though the effectiveness of specific interventions cannot be assessed in this study.

Surgical considerations

Planned surgical intervention was documented for 65% of patients; 42% completed surgery during follow-up. Elevated Wilkins scores (mean 9.2 ± 2.1) and coexisting severe regurgitation limited percutaneous approaches in many cases. Severe pulmonary hypertension (82%) represents a well-established perioperative risk factor. Surgical completion rates and outcomes are described without a comparator; optimal surgical timing cannot be concluded from these data.

Global health implications and future directions

These findings illustrate the ongoing burden of multi-valvular RHD at two Delhi tertiary centers and highlight patterns relevant to public health planning: high rates of missed rheumatic fever diagnosis (only 23% had documented childhood RHD), predominance of lower socioeconomic strata (62%), and seasonal variation in complication profiles. Future analytical research should prospectively define primary outcomes, employ multivariable regression to identify independent predictors of complications and mortality, evaluate comparative treatment strategies, and follow patients systematically over time.

Study limitations

Most importantly, this study used only descriptive statistics. No inferential testing, regression modeling, survival analysis, or multivariable adjustment was performed. Consequently, this study cannot identify independent predictors of complications or mortality, evaluate whether subgroup differences are statistically significant, or establish causal relationships. All findings are hypothesis-generating observations requiring prospective analytical confirmation.

Second, a single-center retrospective design at two specialized academic referral centers in Delhi introduces referral bias toward more severe and complex cases. Complication rates likely overestimate those of the broader multi-valvular RHD population. Generalizability to community hospitals or other geographic regions is limited.

Third, the ≥7-day inclusion criterion may have introduced selection bias by excluding milder cases (early discharge) and rapidly fatal cases (death before day 7), limiting the representativeness of the full severity spectrum.

Fourth, only 67% had complete six-month follow-up data, potentially introducing selection bias in outcome estimates. Clinic-based follow-up may have systematically missed patients with limited healthcare access.

Fifth, retrospective design limits data quality to documentation in medical records. Recall-dependent variables (childhood rheumatic fever history, symptom duration) are likely underreported. Minor echocardiographic measurement variability across operators and equipment over two years cannot be excluded.

Finally, resource constraints in the Delhi public hospital setting may have influenced treatment decisions in ways that limit applicability to better-resourced healthcare systems. 

## Conclusions

This descriptive analysis of 385 patients with severe multi-valvular RHD at Lal Bahadur Shastri Hospital and Dr. Ram Manohar Lohia Hospital characterizes a clinically complex population presenting with advanced hemodynamic compromise. The predominant pattern of severe mitral stenosis with concurrent regurgitation, tricuspid involvement, and pulmonary hypertension created challenging management scenarios requiring multidisciplinary care. Key observed frequencies include atrial fibrillation (58%), severe pulmonary hypertension (82%), and healthcare-associated infections (23%). Preserved left ventricular systolic function was observed in a substantial proportion (mean LVEF 56.3%); this observation may suggest a window of opportunity for timely surgical intervention, but this hypothesis requires prospective analytical confirmation and cannot be concluded from descriptive data alone. 

In-hospital mortality was 3.4% and six-month mortality 7.2% (among those with available follow-up). These are outcomes of this specific hospitalized cohort and should not be generalized to the broader multi-valvular RHD population. Future research should employ prospective cohort designs with pre-specified hypotheses, multivariable risk modeling, standardized surgical timing assessments, and systematic follow-up protocols to translate these descriptive observations into evidence-based management guidance.
